# Effect of the Plasmid-DNA Vaccination on Macroscopic and Microscopic Damage Caused by the Experimental Chronic *Trypanosoma cruzi* Infection in the Canine Model

**DOI:** 10.1155/2013/826570

**Published:** 2013-09-19

**Authors:** Olivia Rodríguez-Morales, Silvia C. Carrillo-Sánchez, Humberto García-Mendoza, Alberto Aranda-Fraustro, Martha A. Ballinas-Verdugo, Ricardo Alejandre-Aguilar, José Luis Rosales-Encina, Maite Vallejo, Minerva Arce-Fonseca

**Affiliations:** ^1^Department of Molecular Biology, Instituto Nacional de Cardiología “Ignacio Chávez”, Juan Badiano No. 1, Col. Sección XVI, Tlalpan, 14080 Mexico City, DF, Mexico; ^2^Department of Pathological Anatomy, Instituto Nacional de Cardiología “Ignacio Chávez”, Juan Badiano No. 1, Col. Sección XVI, Tlalpan, 14080 Mexico City, DF, Mexico; ^3^Department of Parasitology, Escuela Nacional de Ciencias Biológicas del I.P.N., Prolongación de Carpio y Plan de Ayala, Col. Sto. Tomás, Miguel Hidalgo, 11340 Mexico City, DF, Mexico; ^4^Department of Infectomics and Molecular Pathogenesis, Centro de Investigación y de Estudios Avanzados del I.P.N., Avenida Instituto Politécnico Nacional No. 2508, Col. San Pedro Zacatenco, Gustavo A. Madero, 07360 Mexico City, DF, Mexico; ^5^Department of Sociomedical Research, Instituto Nacional de Cardiología “Ignacio Chávez”, Juan Badiano No. 1, Col. Sección XVI, Tlalpan, 14080 Mexico City, DF, Mexico

## Abstract

The dog is considered the main domestic reservoir for *Trypanosoma cruzi* infection and a suitable experimental animal model to study the pathological changes during the course of Chagas disease (CD). Vaccine development is one of CD prevention methods to protect people at risk. Two plasmids containing genes encoding a *trans*-sialidase protein (TcSP) and an amastigote-specific glycoprotein (*TcSSP4*) were used as DNA vaccines in a canine model. Splenomegaly was not found in either of the recombinant plasmid-immunized groups; however, cardiomegaly was absent in animals immunized only with the plasmid containing the *TcSSP4* gene. The inflammation of subendocardial and myocardial tissues was prevented only with the immunization with *TcSSP4* gene. In conclusion, the vaccination with these genes has a partial protective effect on the enlargement of splenic and cardiac tissues during the chronic CD and on microscopic hearth damage, since both plasmids prevented splenomegaly but only one avoided cardiomegaly, and the lesions in heart tissue of dog immunized with plasmid containing the *TcSSP4* gene covered only subepicardial tissue.

## 1. Introduction

American trypanosomiasis, also known as Chagas disease (CD), is a chronic and potentially fatal disorder, now affecting 10 million people in Latin America [1]. It is one of the major endemic problems in Latin America and ranks as the third most important parasitic disease in the world after malaria and schistosomiasis [2]. An estimate of the number of *T. cruzi* infections by states in Mexico was made that indicated that the calculated number of potentially affected people is 5.5 million [3]. Several studies describing the seroprevalence in dogs of this country have been conducted and importantly demonstrated a direct correlation with the seropositivity in humans. The seroprevalence in the dog population ranges between 1.6% and 21% depending on the geographical region [4–8] suggesting that dogs may be domestic reservoir hosts and help to maintain human transmission of *T. cruzi *when dogs and humans cohabit with vector insects, creating thus a public health problem [9].

 Dogs with CD develop diffuse chronic myocarditis with histological and electrocardiographic changes that are also found in humans [10–19]. Prevention of *T. cruzi* transmission is mainly through the control of vector populations, control of blood transfusion and organ transplantation, and monitoring children from chagasic mothers. However, it is necessary to consider other alternatives, such as the development of vaccines that would be key to improve the control of CD. The development and establishment of regulatory immunization schemes have enabled controlling successfully and even eradicating many diseases worldwide. DNA vaccination represents a promising strategy to attempt to prevent many conditions associated with virulent microorganisms, including obligate and facultative intracellular parasites whose ability to reside within the host cells sustains their resistance to the host's defense mechanisms for longer periods [20]. 

 In previous studies in a murine model, *TcSP *and *TcSSP4 *genes immunizations, which were encoding the *Trypanosoma cruzi* TcSP and TcSSP4 proteins, respectively, were found to be effective in inducing antibodies that were increased after the boost. Protection during the acute phase of the disease was partial: *TcSSP4* and *TcSP* gene-immunized/experimentally infected mice showed a 64% and a 73% reduction in parasitemia, respectively. Additionally, survival was 75% in *TcSSP4 *gene-immunized/infected mice and 100% in those immunized with *TcSP *gene and then infected. These studies demonstrated protection during the chronic phase of the disease as revealed by the histological analysis showing that immunization diminishes cardiac damage caused by the H8 strain of *T. cruzi* (MHOM/MX/1992/H8 (*T. cruzi*)) [21–23].

More recently, the clinical and cardiac levels of protection induced by the expression to *T. cruzi *genes encoding the TcSP and TcSSP4 proteins in the canine model were reported. Plasmid DNA vaccination with these *T. cruzi* genes induced a moderate level of protection in the immunized dogs because avoided halted the symptomatic progression to severe heart conduction abnormalities among other effects [24]. 

 Taking advantage of the great similarity between the pathologies developed by chagasic patients and *T. cruzi* infected dogs, the present study explores the role of the protective effect of *TcSP *and *TcSSP4 *gene immunization in Beagle dogs on cardiac and splenic pathological consequences of CD by macro- and microscopic damage findings in order to contribute with new insights about vaccine candidates by reducing infectiousness of the main domestic reservoir of the parasite, as well as by improving the care of companion animals that are increasingly been detected as infected by *T. cruzi*.

## 2. Materials and Methods

### 2.1. Animals

Thirty-five male and female Beagle dogs aged 16 (±1) month, and weighing 14.17 (±3.8) kg were used; all animals had been immunized against canine distemper, canine parvovirus, coronavirus, hepatitis, leptospirosis, canine infectious tracheobronchitis, and rabies, and intestinal deworming. All of the experimental animals were negative for the standardized enzyme-linked immunosorbent assay (ELISA) [25] used to diagnose CD in dogs prior to the start of the study and were healthy, which confirmed the absence of CD before any manipulation. 

Animal handling followed the established guidelines of the International Guiding Principles for Biomedical Research involving Animals and the Norma Oficial Mexicana: Technical Specifications for the Care and Use of Laboratory Animals [26]. Dogs were euthanized in accordance with the Norma Oficial Mexicana: Humane Slaughter of Domestic and Wild Animals [27]; the experimental protocol was approved by the Bioethics Committee of the Instituto Nacional de Cardiología, Ignacio Chávez.

### 2.2. Immunization and Challenge

The animals were divided randomly into 5 groups (*n* = 7). The dogs were immunized with 500 *μ*g DNA dissolved in 500 *μ*L physiological saline solution (SS). The two plasmid constructs (pBCSP and pBCSSP4 containing the *TcSP* and *TcSSP4 *genes, resp.) were generated and characterized as described previously [24]. The experimental dogs of each group were immunized with *TcSP* gene (pBCSP plasmid), *TcSSP4 *gene (pBCSSP4 plasmid), pBK-CMV (empty cloning vector), or mock-immunized with SS twice at 2-week intervals by intramuscular injection; 15 days after the last immunization, these four groups were infected with a well-characterized Mexican *T. cruzi* Ninoa strain (MHOM/MX/1994/Ninoa (*T. cruzi I*)) [23, 28], by intraperitoneal injection with 500,000 metacyclic trypomastigotes per animal that were obtained from urine and feces of triatomines and resuspended in SS. The animals in the healthy control group were not subjected to any experimental procedure. The experimental *T. cruzi *infection was confirmed microscopically (parasitemia) in all infected groups by examining freshly isolated blood samples collected every third day observing from 200 to 400 parasites/mL as limit of detection intermittently between day 22 and day 55 postinfection. All animals were monitored clinically by general physical examinations and electrocardiographics studies. A serological monitoring (ELISA and indirect immunofluorescence as confirmatory test) was also performed in pBK-CMV plasmid-immunized and SS mock-immunized/infected dogs [24] in order to evaluate the establishment of infection by the presence of antibodies anti-*T. cruzi*.

### 2.3. Electrocardiograms (EKGs)

Once CD chronic phase had been determined by serology and electrocardiographic studies at six months postinfection [24], the last EKGs (Schiller, AT-1, USA) for each animal were obtained (previous to the euthanasia, at 11 months after inoculation). No chemical restraint was employed. Three bipolar standard leads (I, II, and III), three augmented unipolar limb leads (aVR, aVL, and aVF), and four unipolar precordial thoracic leads (CV_5_RL, CV_6_LL, CV_6_LU, and V_10_) were recorded. The voltage was standardized at 1 mV/cm, a paper speed of 50 mm/s was used, and electrocardiographic tracings were analyzed according to published data in canine and feline cardiology [29, 30]. 

### 2.4. Euthanasia

Dogs were euthanized at chronic phase (11 months after infection) using sodium pentobarbital (Barbithal, Holland Animal Health, Mexico) as a general anesthetic at a dose of 30 mg/kg applied intravenously, and then a lethal dose of intravenous 15% potassium chloride was administered. 

### 2.5. Macroscopic Evaluation

Prior to euthanizing, weight (Bascule Inpros S.A. de CV, Mexico) was obtained and the heart and the spleen were collected during necropsy and also were weighed (Scout Pro, Ohaus, Mexico). Cardiomegaly and splenomegaly were evaluated by determining the heart and spleen indices (organ weight/total body weight × 100), respectively, and also by inspecting for macroscopic alterations. Cardiomegaly and splenomegaly were considered present in the animals when the organ index was significantly higher than that observed in healthy non-infected animals [31]. 

### 2.6. Histology

Several tissues such as spleen, skeletal muscle, esophagus, ileum, colon, and heart muscle were analyzed. Tissues sections were fixed in 10% buffered formalin solution for 24 h, dehydrated in absolute ethanol, cleared in xylene, and embedded in paraffin for histological examination. Sections (5 *μ*m) were stained with hematoxylin and eosin and evaluated by light microscopy (Carl Zeiss, K7, Germany). Three different sites of heart walls from the top, middle, and bottom of the tissue were analyzed: subepicardium, myocardium, and subendocardium. Images were obtained through a Bio-Doc-It Imaging System image analyzer (UVP, LLC, USA). The severity of inflammation in affected tissue sections was scored on a scale from 1 to 4. A score of 1 indicated one or less foci of inflammatory cells/field (400x); 2, more than one inflammatory foci/field; 3, generalized coalescing of foci of inflammation or disseminated inflammation with minimal cell necrosis and retention of tissue integrity, and 4, diffuse inflammation, with severe tissue necrosis, interstitial edema, hemorrhage, and loss of tissue integrity.

### 2.7. Statistical Analysis

Heart and spleen indices and histological data were analyzed by the Kruskal-Wallis test (SPSS software, version 13.0). Heart and spleen indices data from all vaccinated groups (including the mock-immunized/infected group as positive control for infection) were compared among themselves and between the non-infected group (healthy control). The histological data from all vaccinated groups were compared among themselves and between the mock-immunized/infected group (control for infection). The inflammation 1 to 4 scores were converted to logarithms (base 10) after 1 was added to each score to correct for 0 values [32], and the group means were compared with the statistical test. For all cases, differences were considered as significant when *P* < 0.05. 

## 3. Results

### 3.1. DNA Vaccination with *T. cruzi* Genes Halted the Progression to Severe Heart Electrical Conduction Abnormalities

The survival rate in all experimental groups was 100%. Before the death by euthanasia, the dogs immunized with pBK-CMV empty plasmid or SS mock-immunized exhibited more severely altered electrocardiographic features, and the number of affected dogs was also higher in contrast with the recombinant plasmids-immunized animals (Table 1). 

A P-R interval longer (0.14 s) than normal (0.12 s), associated with atrioventricular (AV) block, was present in 2 dogs (29%) immunized with the *TcSP *gene. One animal (14%) vaccinated with the *TcSSP4 *gene exhibited features that are consistent with microscopic intramural myocardial infarctions (Table 1). In a previous EKG (6 months postinfection) this dog had shown recordings associated with myocardial infarction and left bundle branch block (BBB) [24].

Reversal in polarity of the T wave on serial EKGs was present in 6/7 dogs (86%) of the pBK-CMV empty plasmid-immunized/infected group and in 3/7 dogs (43%) of the SS mock-immunized/infected group. This abnormality was combined with other disturbances suggesting left ventricle enlargement that could be associated to a dilated form of cardiomyopathy caused by a primary myocardial disease such as CD. Altered T wave, QRS complexes, and S waves suggesting right BBB were present in one dog (14%) from the pBK-CMV-immunized/infected group (Table 1). 

Electrocardiographic features associated with ventricular premature complexes were present in 14% of the dogs (1/7) of the SS mock-immunized/infected group. Additionally, disturbances in R wave and electrical alternans that suggested pericardial effusion were also exhibited by two dogs (29%) of this group. Finally, abnormal S-T segment, R wave, and S wave were found in 29% of the animals (2/7) of the SS mock-immunized/infected group, which suggested myocardial infarction and/or pericarditis and right ventricular enlargement (Table 1).

### 3.2. DNA Vaccination with *T. cruzi* Genes Prevents Spleen but Not Heart Damage during the Chronic Stage of the Disease

**Figure 1:**
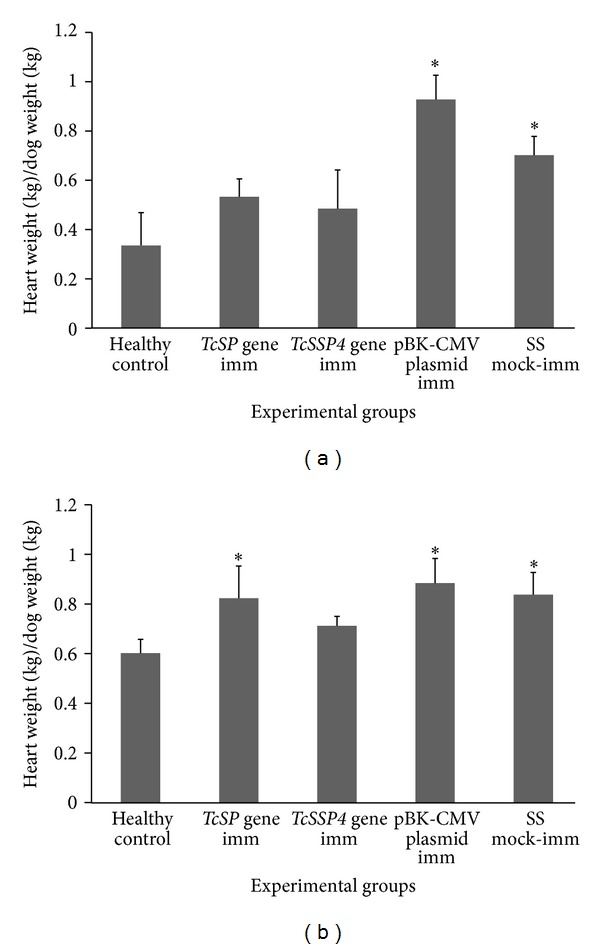
Splenomegaly and cardiomegaly during chronic stage of infection with *Trypanosoma cruzi* Ninoa strain in Beagle dogs. Splenomegaly and cardiomegaly were calculated by the mean spleen (a) and the mean heart (b) indices, respectively (±S.D.). Differences were considered as significant when *P* ≤ 0.05 (Kruskal-Wallis test among immunized/infected groups (including the mock-immunized/infected animals) and healthy non-infected animals was performed). imm = immunization.

The pBK-CMV empty cloning vector-immunized/infected and the SS mock-immunized/infected groups showed splenomegaly (Figure 1(a)) and cardiomegaly (Figure 1(b)) during the chronic phase of the disease as revealed by the mean spleen and heart indices, respectively, as compared with the healthy control group. 

DNA vaccination with the *TcSSP4* and *TcSP* genes prevented splenic tissue damage (Figure 1(a)) during the chronic phase of the infection with *T. cruzi* without differences between them as evidenced by similar spleen index in dogs immunized with each of both genes to that obtained from the healthy non-infected control animals. The dogs immunized with *TcSP *gene (pBCSP plasmid) and the empty plasmid showed heart indices similar to those of SS mock-immunized/infected group (Figure 1(b)), with a significant difference from the healthy non-infected control group, suggesting cardiomegaly, and without differences between themselves. On the contrary, the heart index of the *TcSSP4 *gene-(pBCSSP4 plasmid) immunized/infected group was not different from that of the healthy non-infected control animals (Figure 1(b)), suggesting that probably the *TcSSP4* gene has a partial protective role on cardiac pathological consequences of CD that lead to cardiomegaly. 

### 3.3. Other Macroscopic Alterations in Dogs with Chronic CD

The lesions found in 71% of dogs (5/7) of the SS mock-immunized/infected group and in 86% of animals (6/7) immunized with pBK-CMV empty cloning vector were whitish areas in the heart of fibrous consistency, abundant pericardial fluid, and white areas in the spleen (Table 2). One infected dog (14%) immunized with pBK-CMV plasmid showed ascites, megaesophagus, left ventricular hypertrophy, thinning of the right ventricular wall, and severe tricuspid endocarditis (Table 2). In addition, one dog (14%) of the *TcSSP4* gene-(pBCSSP4 plasmid) immunized/infected group exhibited a heart with adhesions in the trachea and pericardium; splenic thickened walls; and white areas in the spleen and heart (Table 2).

### 3.4. Histopathologic Analysis

Although several tissues were analyzed (spleen, skeletal muscle, esophagus, ileum, colon and heart muscle), the microscopic findings of *T. cruzi-*induced damage in DNA immunized and infected dogs were limited exclusively to heart tissue. No evidence of amastigotes' nests was observed in any tissue analyzed of all experimentally infected dogs. 

**Figure 2:**
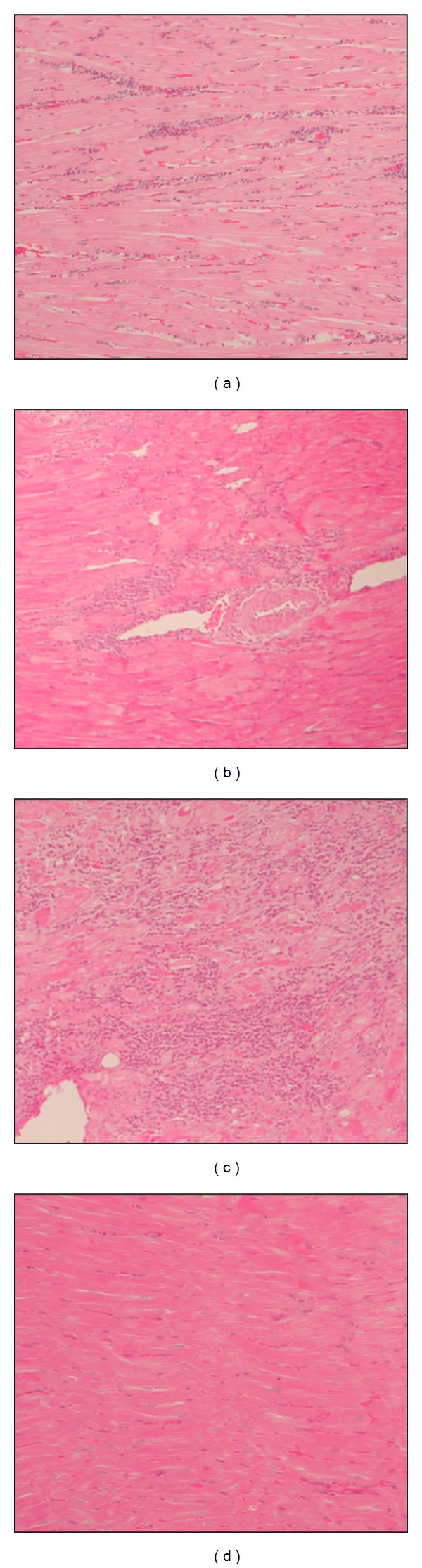
Histological cardiac sections from Beagle dogs DNA-immunized and infected with Ninoa strain of *T. cruzi. *Representative micrographs are shown. (a) Score 1 (1 or less foci of inflammatory cells/field) from a dog immunized with *TcSSP4 *gene (pBCSSP4 plasmid); (b) score 2 (>1 inflammatory foci/field) from a dog immunized with *TcSP *gene (pBCSP plasmid); (c) score 3 (generalized coalescing of foci of inflammation or disseminated inflammation with minimal cell necrosis and retention of tissue integrity) from a dog mock-immunized with SS, and (d) score 0 (without foci of inflammation) from a healthy/uninfected dog. Hematoxylin and eosin stain; 10x magnification.

Similar microscopic changes of sections of heart tissue were found in all DNA immunized/infected and SS mock-immunized/infected groups, including inflammatory infiltrates with mononuclear and polymorphonuclear cells and without parasitism in this tissue (Figure 2). Lymphocytes were the predominant cell infiltrates along with a few histiocytes and neutrophils. Alterations related to heart inflammation were identified in 100% of the dogs immunized with *TcSSP4* gene (pBCSSP4 plasmid), pBK-CMV, and inoculated with SS groups, whereas in the *TcSP* gene-(pBCSP plasmid) immunized/infected animals abnormalities were found in 71% (5/7). However, the extent of inflammation of the heart tissue during the chronic phase of infection was reduced in dogs immunized with the recombinant plasmids, as evidenced by the significant differences in the inflammatory lesion scores between subendocardial tissues from dogs immunized with recombinant plasmids and those immunized with the pBK-CMV empty cloning vector or SS mock-immunized (Table 3), as well as between myocardial tissues from *TcSSP4* gene-(pBCSSP4 plasmid) immunized/infected dogs and from all other groups (Table 3). In immunized/infected groups (including the mock-immunized/infected group as control for infection) with similar inflammatory lesion scores, no significant differences among themselves were found.

## 4. Discussion

In this study cardiomegaly was a condition present in all dogs infected with *T. cruzi* Ninoa strain as one of the symptoms of heart dysfunction in chronic CD. Experimental studies indicate that different *T. cruzi *laboratory strains exhibit varying degrees of tissue tropism, and the pattern of inflammation in tissues and organs allowed the classification of *T. cruzi* strains into six discrete typing units (DTUs) according to their genetic background and biodomes. In Mexico, the most of the *T. cruzi *strains that have been genetically analyzed to date belong to the *T. cruzi *I group, such as *T. cruzi* Ninoa strain (MHOM/1994/MX/Ninoa (*T. cruzi I*)), a reference Mexican strain used in many kinds of studies, which has been classified as a low-virulence strain belonging to biodeme 3 and *T. cruzi *I group in accordance with these criteria [28, 33, 34]; it can behave pathogenically in the human host, as described in the murine model and now in the canine model [24, 25, 35]. 

Mortality in the groups of dogs was not observed throughout the experiment, unlike other studies where experimental *T. cruzi* inoculation caused sudden death in some of the infected dogs [10, 11, 17, 32, 36]; however, some of these authors worked with mongrel dogs and with different number of animal samples, with another *T. cruzi* strain or morphologic form of the parasite and different amounts of inoculum, or employed different administration routes. Our experimental canine model of CD has already been standardized in accordance with lethal doses 50 assays to determine an adequate inoculum and achieving the establishment of the acute and chronic phases of CD through the systemic infection (one of the natural routes of infection in dogs, besides of the oral route by ingesting the vector).

Our observations confirmed that DNA-immunization did not abrogate the parasitemia found in all experimental groups during the acute phase of CD; however, the antibodies produced by DNA-immunized dogs mediated the lysis of parasites through the complement system, which had an impact during the acute phase of the illness because parasitemia and parasite load was lower over time in the animals of the vaccinated groups than those nonimmunized dogs [35].

The cardiomegaly, inflammation, and fibrosis were associated to the presence of high *in vitro* infectivity of *T. cruzi* strains. Despite that Ninoa *T. cruzi* is a strain of low virulence, it was possible to find heart and spleen histological changes suggestive of cardiomegaly, myocarditis, and splenomegaly, as reported by other authors [18, 28, 31, 33, 34]. 

Regarding the spleen index, the results show no significant difference in the groups immunized with either *TcSP *gene or *TcSSP4 *gene, unlike the group immunized with the empty cloning vector pBK-CMV, which coincided with splenic indices of SS mock-immunized/infected dogs. This event indicates that infection with the *T. cruzi *Ninoa strain in the canine model induced splenomegaly in CD chronic phase similarly to other more virulent strains in humans and dogs [18, 37], and the DNA vaccination with parasite *TcSP* and *TcSSP4* genes showed a significant amelioration of splenic pathology, as assessed by the splenic index. This finding correlates directly with splenic tissue damage during the chronic stage of the disease. Parasite *TcSP *and *TcSSP4 *genes encode a *trans*-sialidase and an amastigote specific-protein, which are expressed in all forms of the parasite and in trypomastigote to amastigote transition forms, respectively, stimulating a strong humoral and cellular response during BALB/c mice infection and preventing pathological manifestations of chronic CD [21, 22]. 

Lesions observed in chronic chagasic cardiomyopathy (CCC) frequently produce electrocardiographic alterations and affect cardiac function. The abnormalities recorded in the EKGs agreed with the cardiomegaly found in the infected dogs and the *TcSP *gene-immunized/infected group, this is consistent with reports in studies with Beagle dogs infected with other *T. cruzi* strains, where mild splenomegalies and severe cardiomegalies were observed in chronic CD [18, 31]. In contrast, the dogs immunized with the *TcSSP4* gene at the same dose and then infected exhibited a heart index similar to that of the healthy non-immunized group, and probably *Tc*SSP4, the amastigote specific protein, encoded by the *TcSSP4* gene, had a partial protective role during CCC likely because this gene induces immunity against the stage-specific surface antigen present on the intracellular form. Further studies of immunizations with the two plasmids in combination are required to determine if there is a possible synergistic effect. 

In some reports that used mongrel dogs for experimental CD cardiomegaly, chronic myocarditis with variable reduction levels of cardiac muscle, fibrosis, and adipose tissue replacement was the most frequent findings [10, 13, 14].

Other macroscopic findings at necropsy in some dogs are consistent with reports that registered macroscopic and microscopic injuries, such as slight right ventricle dilation and infarcted areas in cardiac tissue, different degrees of lymphoplasmacytic inflammation in myocardium [36], diffuse fibrous cardiopathy [11], chronic focal and discrete myocarditis, whitish thickening in the form of plaque in the right atrium (chronic productive epicarditis), chronic productive perisplenitis [38], fibrosis partially or completely interrupting the path of muscle bundles, cardiomegaly, heart with a globus shape, hydropericardium [13], dilated cardiomyopathy (biventricular dilation), focal and diffused myocarditis, round shaped hearts, and epicardial hemorrhages [19].

No amastigote nests were found in any heart sections obtained from immunized/infected or mock-immunized/infected dogs with chronic CD; this is consistent with other studies in dogs [12, 13, 38]. Parasites in tissue fragments of dogs, mice, and humans have only been found during the acute stage of CD, but not during the chronic stage [19, 37, 39]; however, effective immunological control of inflammation during the chronic phase of infection resulted in reduced lymphocyte infiltrates by vaccination with the *TcSSP4 *gene. 

## 5. Conclusions

*T. cruzi* Ninoa strain infection induces splenomegaly and cardiomegaly, and DNA vaccination with both *TcSP* and *TcSSP4* genes prevents splenic but not cardiac tissue damage during chronic CD. Immunization with the *TcSSP4* gene (pBCSSP4 plasmid) had a partial protective role avoiding cardiomegaly and microscopic heart damage since the dogs of this group showed heart indices similar to those of healthy animals, and the microscopic lesions did not reach myocardial or subendocardial tissues; therefore, DNA vaccination could be an approach to reduce the severity of chronic *T. cruzi* infection. 

## Figures and Tables

**Table 1:** Number of animals with presence of major cardiac anomalies (by EKG) in DNA-immunized dogs with chronic experimental Chagas disease.

Group	Dogs/*n* (%)	Suggested pathological conditions by EKG features found [29, 30]
*TcSP *gene (pBCSP plasmid)	2/7 (29%)	AV block
	1/7 (14%)	Left ventricular enlargement
*TcSSP4 *gene (pBCSSP4 plasmid)	1/7 (14%)	MIMI
	1/7 (14%)	Second-degree AV block
pBK-CMV (empty plasmid)	6/7 (86%)	Most often abnormal when found on serial EKG and combined with other disturbances
	4/7 (57%)	Left ventricle enlargement
	1/7 (14%)	Right BBB
SS (mock-immunized)	3/7 (43%)	Most often abnormal if it is found on serial EKG and combinedwith other disturbances
	1/7 (14%)	VPC
	2/7 (29%)	Pericardial effusion
	2/7 (29%)	Myocardial infarction and/or pericarditis and right ventricular enlargement

EKG: electrocardiogram.

AV: atrioventricular.

MIMI: microscopic intramural myocardial infarctions.

BBB: bundle branch block.

SS: physiologic saline solution.

VPC: ventricular premature complexes.

**Table 2:** Other macroscopic alterations in DNA-immunized dogs with chronic experimental Chagas disease.

Macroscopic pathological features at 11 months after infection (at the time of euthanizing)	Groups	Dogs/*n* (%)
Whitish areas in heart of fibrous consistency Abundant pericardial fluid Whitish areas in spleen	SS (mock-immunized) and pBK-CMV (empty plasmid)	5/7 (71%) 6/7 (86%)
Ascites Megaesophagus Left ventricular hypertrophy Thinning of the right ventricular wall Severe tricuspid endocarditis	pBK-CMV (empty plasmid)	1/7 (14%)
Heart with adhesions in trachea and pericardium Splenic thickened walls Whitish areas in spleen and heart	*TcSSP4 *gene (pBCSSP4 plasmid)	1/7 (14%)

**Table 3:** Inflammatory lesion (lymphocyte infiltrates) scores from three different sections of heart tissues of DNA-immunized dogs with chronic experimental Chagas disease.

Site	*TcSP *gene (pBCSP plasmid)	*TcSSP4 *gene (pBCSSP4 plasmid)	pBK-CMV (empty plasmid)	SS (mock-immunized)
Subepicardium	0.800 ± 0.2*	0.99 ± 0.4*	1.0714 ± 0.4*	1.0 ± 0.0*
Myocardium	0.7429 ± 0.1*	0.5143 ± 0.1	0.7143 ± 0.4*	0.9429 ± 0.4*
Subendocardium	0.1429 ± 0.03	0.2857 ± 0.04	0.7143 ± 0.4*	0.8932.3*

*Differences were considered as significant when *P* ≤ 0.05 (Kruskal-Wallis test among immunized/infected and healthy non-infected control animals, whose values obtained were 0.0, was performed).
